# Ruxolitinib-treated polycythemia vera patients and their risk of secondary malignancies

**DOI:** 10.1007/s00277-021-04647-0

**Published:** 2021-08-31

**Authors:** Rohit Sekhri, Parvis Sadjadian, Tatjana Becker, Vera Kolatzki, Karlo Huenerbein, Raphael Meixner, Hannah Marchi, Rudolf Wallmann, Christiane Fuchs, Martin Griesshammer, Kai Wille

**Affiliations:** 1 University Clinic for Hematology, Oncology, Hemostaseology and Palliative Care, Johannes Wesling Medical Center Minden, University of Bochum, Hans-Nolte-Straße 1, 32429 Minden, Germany; 2 Institute of Computational Biology, Helmholtz Center Munich, Ingolstädter Landstraße 1, 85764 Neuherberg, Germany; 3 Bielefeld University, Universitätsstraße, 25 33615 Bielefeld, Germany

**Keywords:** Ruxolitinib, Polycythemia vera, Secondary malignancy, Cytoreductive therapy, Non-melanoma skin carcinomas

## Abstract

Recently, there has been increased concern about a risk of secondary malignancies (SM) occurring in myelofibrosis (MF) patients receiving ruxolitinib (RUX). In polycythemia vera (PV), on the other hand, only limited data on the risk of SM under RUX treatment are available. To investigate the association between RUX therapy in PV and SM, we conducted a retrospective, single-center study that included 289 PV patients. RUX was administered to 32.9% (95/289) of patients for a median treatment duration of 48.0 months (range 1.0–101.6). Within a median follow-up of 97 months (1.0–395.0) after PV diagnosis, 24 SM occurred. Comparing the number of PV patients with RUX-associated SM (*n* = 10, 41.7%) with the 14 (58.3%) patients who developed SM without RUX, no significant difference (*p* = 0.34, chi square test) was found. No increased incidences of melanoma, lymphoma, or solid “non-skin” malignancies were observed with RUX (*p* = 0.31, *p* = 0.60, and *p* = 0.63, respectively, chi square test). However, significantly more NMSC occurred in association with RUX treatment (*p* = 0.03, chi-squared test). The “SM-free survival” was not significantly different by log rank test for all 289 patients (*p* = 0.65), for the patients (*n* = 208; 72%) receiving cytoreductive therapy (*p* = 0.48) or for different therapy sequences (*p* = 0.074). In multivariate analysis, advanced age at PV diagnosis (HR 1.062 [95% CI 1.028, 1.098]) but not administration of RUX (HR 1.068 [95% CI 0.468, 2.463]) was associated with an increased risk for SM (*p* = 0.005). According to this retrospective analysis, no increased risk of SM due to RUX treatment could be substantiated for PV.

## Introduction

Polycythemia vera (PV) is one of the three classic *BCR/ABL*-negative myeloproliferative neoplasm (MPN) and is characterized by an increased red blood cell mass induced by driver mutations in the *JAK2* gene [1]. Cardiovascular events and transformations to secondary myelofibrosis or acute leukemia are recognized causes of shortened life expectancy in PV patients [2–4]. In recent years, the development of secondary malignancies (= SM) has come into focus as a further risk factor for this increased mortality [5]. Several retrospective studies reported an increased incidence of SM in MPN patients compared to the healthy population, but the exact underlying pathogenesis is still controversial [6–8].

Ruxolitinib (RUX), a selective JAK1/2-inhibitor, was approved by the FDA in 2012 for the treatment of patients with primary or secondary myelofibrosis (MF) with symptomatic splenomegaly and/or debilitating disease-related symptoms. In 2015, approval followed for PV patients with inadequate response or intolerance to hydroxyurea (HU) based on the data of the two pivotal studies “RESPONSE 1” [9] and “RESPONSE 2” [10].

However, concerns have recently emerged about an association between RUX treatment and an increased risk of SM. In 2018, Porpaczy et al. [11] reported a 16-fold increased risk of aggressive lymphoma associated with JAK1/2-inhibitor treatment in 126 MPN patients, diagnosed mainly with MF, in a retrospective study from two academic centers. However, another retrospective multicenter study by Maffioli et al. [14], which included 219 RUX-treated patients with primary and secondary MF, did not report an increased risk of lymphoma compared to RUX-naive MF patients. In a subsequent retrospective multicenter study by Mora et al. [12], which included 2233 secondary MF patients, no association was also observed between treatment with JAK1/2-inhibitors and an increased risk of SM, with the exception of the occurrence of non-melanoma skin cancer (NMSC).

Compared to the aforementioned data for MF, there are only rare data on the risk of SM in PV patients under RUX treatment. In 2020, Kiladjian et al. [24] published the 5-year follow-up data of the “RESPONSE 1” study and reported an increased number of SM in the group of patients initially treated with RUX compared to the best available therapy (BAT) and the crossover population. It is noteworthy that two patients died under RUX treatment due to SM (one gastric adenocarcinoma and one malignant neoplasm of the lung). Preliminary data from the “RESPONSE 2” study presented by Passamonti et al. [25] showed an increased rate of NMSC in the RUX-treated and crossover populations compared to BAT. One patient in the RUX arm died due to SM (metastatic melanoma). Therefore, the aim of our retrospective, single-center study of 289 PV patients was to obtain and evaluate further “real-world” data on the presumed association between RUX treatment and an increased risk of developing SM in patients with polycythemia vera.

## Patients and methods

The data of all PV patients presenting regularly at our university hospital were collected. The patients gave their consent to the data collection. All patients had a PV diagnosis according to the WHO 2016 criteria [16]. Our main objective was to investigate a potential increased risk of SM in PV patients with RUX treatment. The enrollment period of this analysis started at 14-May-2013. The date of the last data collection (“data cut-off”) was 01-December-2020. The study was approved by the institutional review board of our center.

In total, data from 289 PV patients were included in this retrospective study. All of them were JAK2 V617F mutated. Two hundred seventy-two patients (94.1%) were observed only in our retrospective analysis. Seventeen of the 289 patients (5.9%) were additionally included in the prospective “RESPONSE 1” or “RESPONSE 2” studies. Follow-up time was defined as the time from PV diagnosis until the last “data cut-off” (01-December 2020), SM diagnosis, transformation to a secondary MF/acute leukemia or to death, whichever came first. The data was recorded in an electronic system. In brief, the following information was recorded for each patient: demographic data, mutational profile, spleen size, presence of constitutional symptoms at time of MPN diagnosis and transformation to AML and/or secondary MF. In addition, patient data were collected such as antiplatelet or anticoagulant medications, and treatment with PV-specific cytoreductive therapy (type, date of onset, date of discontinuation, and reason for discontinuation) were recorded. The following drugs were defined as PV-specific cytoreductive therapies: hydroxyurea (HU), ruxolitinib (RUX), interferon alpha (IFN), anagrelide (ANA), and busulfan. Phlebotomies and/or administration of low-dose acetylsalicylic acid (ASA) were performed according to the “DGHO”-guidelines [17] (DGHO = German Society for Hematology and Oncology) and were not defined as PV-specific cytoreductive therapies.

The “PV survival score” by Tefferi et al. [18] was used at PV diagnosis to estimate the probability of survival of each patient. For this score, the following data were recorded and assigned the corresponding points: age at PV diagnosis (≥ 67 years = 5 points; 55–67 years = 2 points), leukocyte count at PV diagnosis (≥ 15 × 10^9^/l = 1 point), and venous thrombosis before/at PV diagnosis (any event = 1 point). Based on the score, the following risk groups were defined: low-risk (0 points), intermediate-risk (1 or 2 points), and high-risk (≥ 3 points).

The next step was to identify PV patients who developed SM at the time of PV diagnosis or afterwards. The diagnosis of SM was only accepted if it was confirmed by objective methods. A positive histological result (after taking a biopsy) was mandatory. Further staging examinations such as ultrasonography, CT, PET CT, or NMR were used.

The diagnosis SM included skin malignancies such as melanomas (MSC), non-melanoma skin malignancies (NMSC), solid malignancies, and hematological malignancies. Transformation to acute myeloid leukemia (AML) or secondary MF was not defined as SM.

Finally, data further characterizing the SM, such as stage, treatment, remission state, and patient outcome, were collected.

## Statistical methods

For continuous variables, median and range were provided. The annual incidence of SM was calculated by dividing the number of events by the total number of patient-years.

For comparison of clinical parameters, the chi square test or the Wilcoxon test were performed. The log-rank-test was used to compare the Kaplan–Meier curves. The Cox regression model was used to account for the effects of multiple variables on SM. For all the analyses, we used the significance level $\alpha =0.05$.

## Results

We included 289 PV patients with a higher proportion of women (58.8%). At the time of PV diagnosis, most patients (52.2%) were between 40 and 60 years of age and were classified as low-risk according to the “PV survival score” (50.5%). The median follow-up time was 97.0 months (range 1.0–395.0) and the median duration of cytoreductive PV therapy 73.5 months (1.0–311.0). Most patients received hydroxyurea (HU, *n* = 185; 64.0%) for a median treatment time of 45.2 months (0.2–289.0), followed by 95 (32.9%) patients with RUX treatment for a median time of 48.0 months (1.0–101.6). The reasons for starting RUX were an intolerance to HU in 58 of the 95 patients (61.1%), 23 (24.2%) patients developed a resistance to HU, in twelve (12.6%) cases, RUX was started based on an individual decision of the attending physician, and another four (4.2%) patients participated in clinical studies.

Cytoreductive therapy except HU or RUX was administered in 23.2% (*n* = 67) of patients for a median time of 38.0 months (1.0–275.0). In detail, interferon alpha was used in 40 of 67 patients (59.7%), anagrelide in 21 (31.3%) and busulfan in six patients (9.0%).

Transformation to secondary myelofibrosis was diagnosed in 46 of 289 patients (15.9%) patients and six (2.1%) developed secondary acute leukemia.

The clinical parameters of the 289 PV patients are summarized in Table 1.

**Table 1:** Clinical characteristics of the 289 PV patients

Male/female – n (%)	119/170 (41.2/58.8)
Median age at PV diagnosis, years (range)	52.1 (11.0–84.4)
- < 40 years, n (%)	59 (20.4)
- 40–60 years, n (%)	151 (52.2)
- > 60 years, n (%)	79 (27.4)
“PV survival score”^a^ at diagnosis, n (%)	
- High risk	59 (20.4)
- Intermediate risk	84 (29.1)
- Low risk	146 (50.5)
Median follow-up time, months (range)	97.0 (1.0–395.0)
Median treatment time on cytoreductive therapy^b^, months (range)	73.5 (1.0–311.0)
Median treatment time on HU^c^, months (range)	45.2 (0.2–289.0)
Median treatment time on RUX^d^, months (range)	48.0 (1.0–101.6)
Median treatment time of cytoreductive therapy^e^ except HU/RUX, months (range)	38.0 (1.0–275.0)
Number of patients receiving HU^c^, n (%)	185 (64.0)
Number of patients receiving RUX^d^, n (%)	95 (32.9)
Number of patients receiving cytoreductive therapy^e^ except HU/RUX, n (%)	67 (23.2)
Patients with a transformation to secondary myelofibrosis, n (%)	46 (15.9)
Patients with a transformation to acute leukemia, n (%)	6 (2.1)

a) “PV survival score” [18]: age at first diagnosis (≥ 67 years = 5 points; 55–67 years = 2 points), leukocyte count at diagnosis (≥ 15 × 10^9^/l = 1 point) and venous thrombosis before or at diagnosis (1 point): low-risk (0 points), intermediate-risk (1 or 2 points), and high-risk (≥ 3 points)

b) ASA/phlebotomies were not defined as “cytoreductive therapy”. All patients received phlebotomies and/or low-dose ASA according to “DGHO”-guidelines [17]

c) *HU* hydroxyurea

d) *RUX* ruxolitinib

e) Cytoreductive therapy except HU or RUX: interferon alpha *n* = 40, anagrelide *n* = 2 1, or busulfan *n* = 6, respectively

During follow-up time, 24 secondary malignancies (SM) occurred in in 24/289 PV patients (8.3%). The median time to development of the 24 SM was 7.2 years (range 0.1–22.9) after PV diagnosis and the median age at SM diagnosis was 69.7 years (range 29.6–89.5). Most of the SM (9/24; 37.5%) occurred in patients with an “intermediate risk” according to the “PV survival score” [18].

Skin cancer was the most prevalent SM subtype (*n* = 13; 54.2%), with eleven (45.8%) non-melanoma skin cancers (NMSC) and two (8.3%) melanoma skin cancers (MSC), respectively. Among other solid “non-skin” cancers (*n* = 9; 37.5%), breast cancer was the frequent subtype (*n* = 4; 16.7%).

Regarding the occurrence of lymphomas, two patients were diagnosed with two lymphomas (one diffuse large B cell lymphoma (DLCBL) and one follicular lymphoma (FL)). DLBCL occurred during RUX treatment. The patient received immune chemotherapy and was in complete remission at the last visit to our center. The follicular lymphoma occurred in a patient without RUX therapy. This patient is alive and the lymphoma is in partial remission at the last visit to our center. Only one patient died during the follow-up period due to SM (a colon carcinoma in a patient without RUX treatment).

Regarding RUX therapy-associated SM, 10/24 (41.7%) SM occurred in ten RUX-treated patients. 8/10 SM were diagnosed during RUX therapy in a median time of 21.3 months after the start of RUX therapy (range 0.3–3.9 years). Two SM occurred in two patients in a median time of 7.2 months after completion of RUX therapy. These two SM were considered to be RUX-associated, because neither patient received cytoreductive therapy between the end of RUX treatment and the development of the two SM. Remarkably, none of the patients with RUX treatment developed SM between PV diagnosis and RUX onset. Fourteen of the 24 SM (58.3%) occurred in patients without RUX therapy. The clinical features of the 24 SM are summarized in Table 2.

**Table 2:** Clinical features, numbers, and types of 24 secondary malignancies (SM)

Type of all 24 secondary malignancies, n (%)	
**Non-melanoma skin cancers (NMSC)**	**11 (45.8)**
- Basal cell carcinoma	6 (25.0)
- Squamous cell cancer	2 (8.3)
- Others^a^	3 (12.5)
**Melanoma skin cancers (MSC)**	**2 (8.3)**
**Solid “non-skin” cancers**	**9 (37.5)**
- Breast cancer	4 (16.7)
- Colon cancer	2 (8.7)
- Other solid cancers^b^	3 (12.5)
**Hematological malignancies**^**c**^	**2 (8.3)**
Median age at SM diagnosis, years (range)	69.7 (29.6–89.5)
Median time between PV diagnosis and SM diagnosis, years (range)	7.2 (0.1–22.9)
Number of SM according to “PV survival score”^d^ at diagnosis, n (%)	
- High risk	8 (33.3)
- Intermediate risk	9 (37.5)
- Low risk	7 (29.2)
Number of RUX-therapy associated SM, n (%)	10 (41.7)
Number of SM occurring in patients without RUX treatment, n (%)	14 (58.3)

a) Merkel cell carcinoma (*n* = 1), Bowen´s disease (*n* = 1), actinic keratosis (*n* = 1)

b) Urothelial carcinoma (*n* = 1), schwannoma (*n* = 1), lung cancer (*n* = 1)

c) Aggressive B cell lymphoma (*n* = 1), indolent B cell lymphoma (*n* = 1)

d) “PV survival score” [18]: age at first diagnosis (≥ 67 years = 5 points; 55–67 years = 2 points), leukocyte count at diagnosis (≥ 15 × 10^9^/l = 1 point) and venous thrombosis before or at diagnosis (1 point): low-risk (0 points), intermediate-risk (1 or 2 points) and high-risk (≥ 3 points)

The incidence rate for the 24 SM was 0.87% per patient/year. Remarkably, the incidence rate for the RUX therapy-associated SM was slightly increased with 1.00% per patient/year compared to the incidence rate for SM in the group without RUX therapy of 0.81% per patient/year.

Of note, another four SM occurred in four PV patients after transformation to secondary myelofibrosis (outside the follow-up time as defined above). Four of these four patients received RUX before SM diagnosis (two before transformation and two thereafter).

A univariate comparison of clinical parameters between the group of PV patients with (*n* = 95) or without (*n* = 194) RUX treatment is shown in Table 3. In the group with RUX treatment, the median follow-up time (*p* = 0.01), the median treatment time on HU (*p* = 0.001), and the median treatment time with cytoreductive therapy except HU (*p* = 0.005) were significantly different.

**Table 3:** Comparison of 289 PV patients treated with (*n* = 95) or without (*n* = 194) ruxolitinib (RUX)

Parameters	PV patients with RUX treatment (*n* = 95)	PV patients without RUX treatment (*n* = 194)	*p*
Male/female, n	42/53	77/117	*p* = 0.54
Median age at PV diagnosis, years (range)	52.9 (17.7–83.5)	51.8 (11.0–84.4)	*p* = 0.13
Median time between PV diagnosis and SM diagnosis, years (range)	7.0 (1.4–22.9)	7.5 (0.1–16.7)	*p* = 0.80
“PV survival score”^a^, n (%)			
- High risk	25 (26.3)	34 (17.5)	*p* = 0.06
- Intermediate risk	22 (23.2)	62 (32.0)	*p* = 0.08
- Low risk	48 (50.5)	98 (50.5)	*p* = 0.90
Median follow-up time, years (range)	9.8 (1.2–29.9)	7.7 (0.1–32.9)	*p* = 0.01*
Patients with secondary malignancies, n (%)	10 (10.5)	14 (7.2)	*p* = 0.34
- With NMSC^c^	7 (7.4)	4 (2.1)	*p* = 0.03*
- With MSC^d^	0 (0.0)	2 (1.0)	*p* = 0.31
- With solid “non-skin” malignancies	2 (2.1)	6 (3.1)	*p* = 0.63
- Lymphoma	1 (1.0)	1 (0.5)	*p* = 0.60
Median age at diagnosis of secondary malignancy, years (range)	71.2 (57.4–89.5)	66.6 (29.6–82.3)	*p* = 0.31
Median treatment time on cytoreductive therapy, months (range)	75.0 (5.6–311.0)	70.6 (1.0–291.0)	*p* = 0.25
Median treatment time on HU^e^, months (range)	22.0 (0.5–254.3)	48.2 (0.2–289.0)	*p* = 0.001*
Median treatment time on cytoreductive therapy^f^ except HU, months (range)	10.9 (1.0–129.0)	45.0 (0.9–275.0)	*p* = 0.005*
Patients with transformation to secondary myelofibrosis, n (%)	14 (14.7)	32 (16.5)	*p* = 0.83
Patients with transformation to acute leukemia, n (%)	2 (2.1)	4 (2.1)	*p* = 1.0
Deaths, n (%)	10 (10.5)	14 (7.2)	*p* = 0.34
Due to secondary malignancy	0	1 (0.5)	n.a.^g^

a) PV survival score” [18]: age at first diagnosis (≥ 67 years = 5 points; 55–67 years = 2 points), leukocyte count at diagnosis ≥ 15 × 10^9^/l (1 point) and venous thrombosis before or at diagnosis (1 point): low-risk (0 points), intermediate-risk (1 or 2 points), and high-risk (≥ 3 points)

b) *NMSC* non-melanoma skin malignancy

c) *MSC* melanoma skin malignancies

d) *HU* hydroxyurea

e) PV-specific cytoreductive therapy except HU: anagrelide, busulfan, or interferon, respectively

f) *n.a*. not applicable

^*^ Statistical significant (*p* < 0.05, chi square and Wilcoxon test)

For the number of patients who developed SM, the difference between the two groups was not statistically significant (*p* = 0.34). Among the SM subtypes, the number of patients with melanoma skin cancer (*p* = 0.31), lymphoma (*p* = 0.60), and solid “non-skin” malignancies (*p* = 0.63) were also not significantly different. However, significantly more patients with RUX treatment developed non-melanoma skin cancer (NMSC) (*p* = 0.03).

All other clinical parameters listed in Table 3 were not statistically different.

**Fig. 1:**
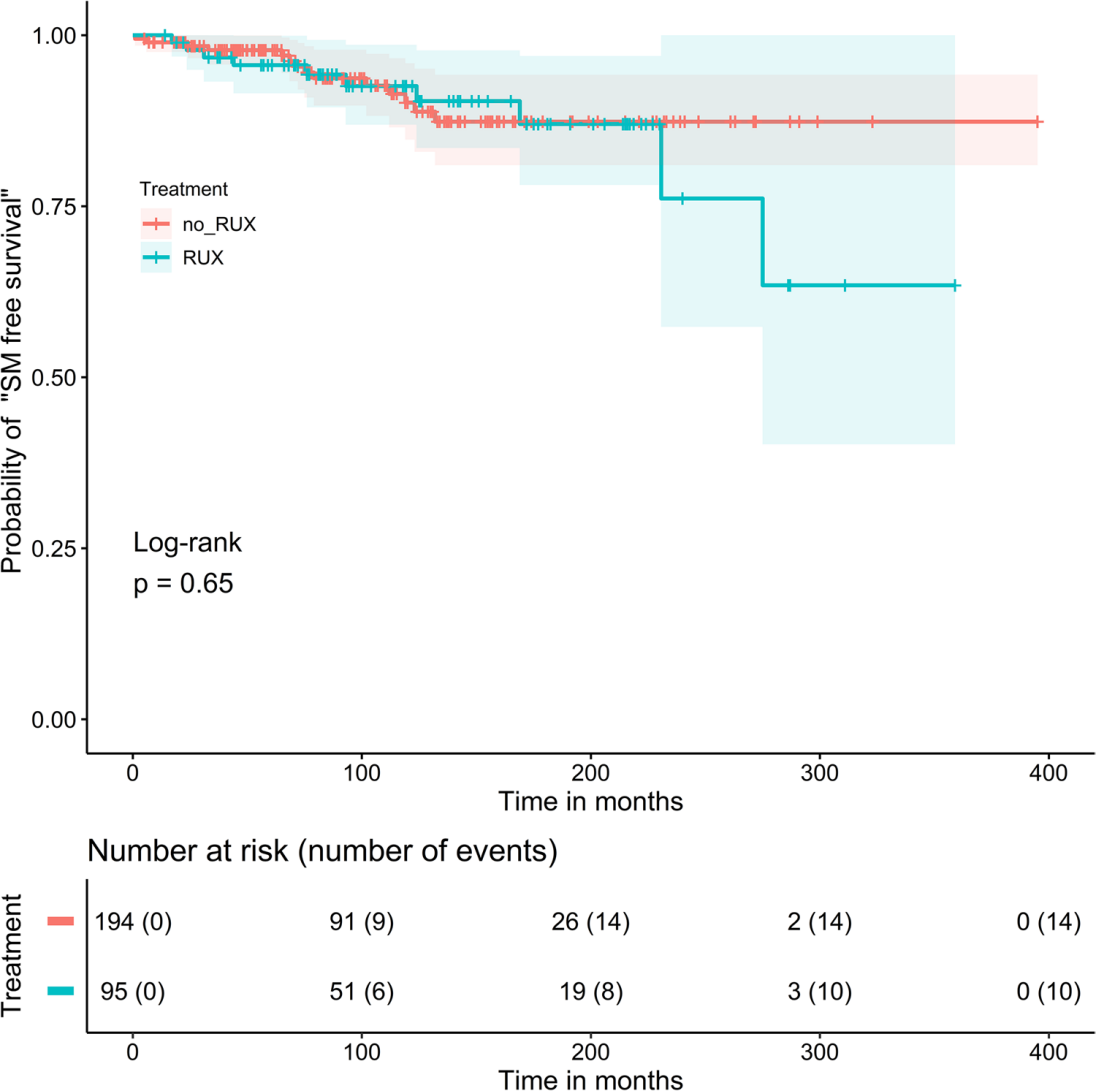
Probability of “secondary malignancy (SM) free survival” in 289 PV patients starting at time of the PV diagnosis: The difference in cumulative probabilities of “SM free survival” in 95 MPN patients treated with ruxolitinib (RUX) (blue curve) or without RUX (*n* = 194, red curve) was statistically not significant (*p* = 0.65; log-rank test)

After comparing the absolute number of SM, a log-rank test was performed that considered the probability of “SM free survival” during follow-up time starting with the time of PV diagnosis in the 289 PV patients. In this analysis, the difference between the patients with RUX treatment (*n* = 95) versus the patients without RUX treatment (*n* = 194) was not statistically different (Fig. 1, *p* = 0.65).

To analyze further covariates that could influence the risk of SM in the 289 patients, we conducted a multivariate Cox regression with the variables age at PV diagnosis, gender, occurrence of venous thrombosis, and administration of ruxolitinib as covariates. The estimators for the covariates can be taken from Table 4. Overall, the model was shown to be significant, i.e., the covariates were appropriate to explain the dependent variable (*p* = 0.005). According to this analysis, only age at PV diagnosis was a significant risk factor for the development of SM (HR 1.062).

**Table 4:** Multivariate Cox regression of the 289 PV patients with cytoreductive therapies with the variables age at PV diagnosis, gender, occurrence of venous thrombosis, and administration of ruxolitinib. The 95% confidence intervals for the estimators are given in parentheses. Only age at PV diagnosis was statistically significant (HR 1.062)

Multivariate COX regression	
**Variable**	**HR** [95% CI]
Age at PV diagnosis	1.062 [1.028, 1.098]*
Gender	1.232 [0.522, 2.908]
Occurrence of venous thrombosis	0.452 [0.105, 1.936]
Administration of ruxolitinib	1.068 [0.468, 2.463]

^*^Statistically significant

**Fig. 2:**
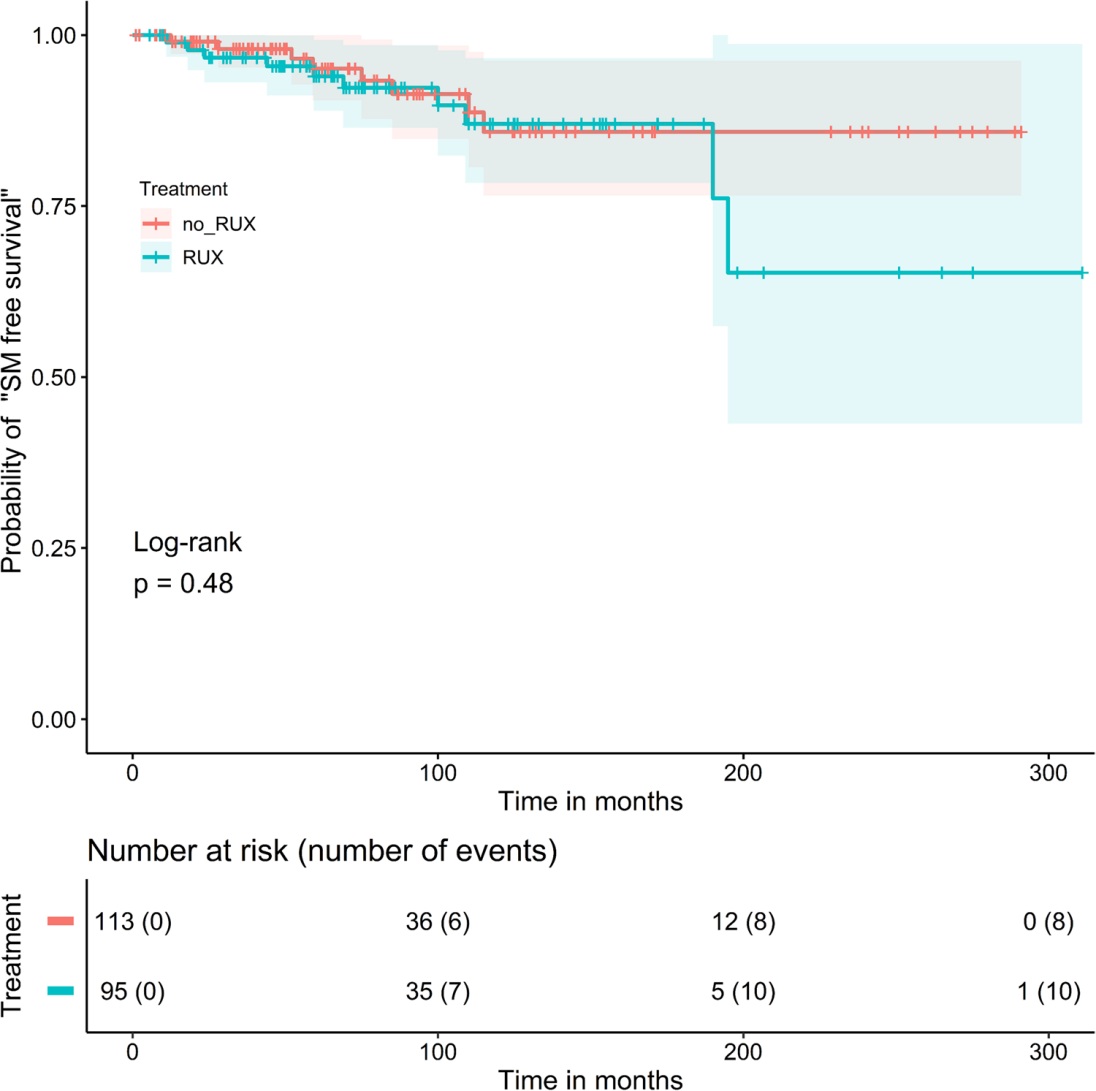
Probability of “secondary malignancy (SM) free survival” in 208 PV patients with cytoreductive treatment starting at time of first drug administration: The difference in cumulative probabilities of the “SM free survival” in 208 MPN patients treated with ruxolitinib (RUX) (*n* = 95, blue curve) or without RUX (*n* = 113, red curve) was not statistically significant (*p* = 0.48; log-rank test)

In a next step, we focused only on patients who received cytoreductive treatments during the follow-up period. Overall, in 208/289 patients (72.0%), cytoreductive therapy was administered. The other 81 patients (28.0%) were treated with ASA/ phlebotomies alone. Regarding the probability of “SM free survival” in the 208 patients with cytoreductive treatment starting at the time of the first drug administration, the difference between the patients with RUX treatment (*n* = 95; 45.7%) versus the patients without RUX treatment (= patients with cytoreductive therapy other than RUX; *n* = 113; 54.3%) was also not statistically different in the log-rank test (Fig. 2, *p* = 0.48).

In addition, the 208 patients with cytoreductive treatments were subdivided into six subgroups with different therapy sequences. In the first group (“HU only”; *n* = 72; 34.6%), only HU was used. In the 2nd group (“RUX only”; *n* = 13; 6.3%), only RUX and in the 3rd group (“IFN only”; *n* = 11; 5.3%), only IFN was used. The 4th group (“HU/RUX”; *n* = 60; 28.8%) includes only patients with the order: first HU, then RUX. The 5th group (“therapy sequences without RUX”; *n* = 30; 14.4%) was treated with a sequence of different cytoreductive drugs other than RUX. Of note, patients with HU only or IFN only were not included in this group. The 6th group (“therapy sequences with RUX”; *n* = 22; 10.6%) includes patients who received a sequence of different cytoreductive drugs including RUX. Of note, patients with the sequence HU followed by RUX or with RUX only were not included in this group.

Remarkably, no SM was observed in the “IFN only” and in the “therapy sequences without RUX” group. Most SM (*n* = 9) occurred in the 4th group; 7/9 were NMSC.

**Fig. 3:**
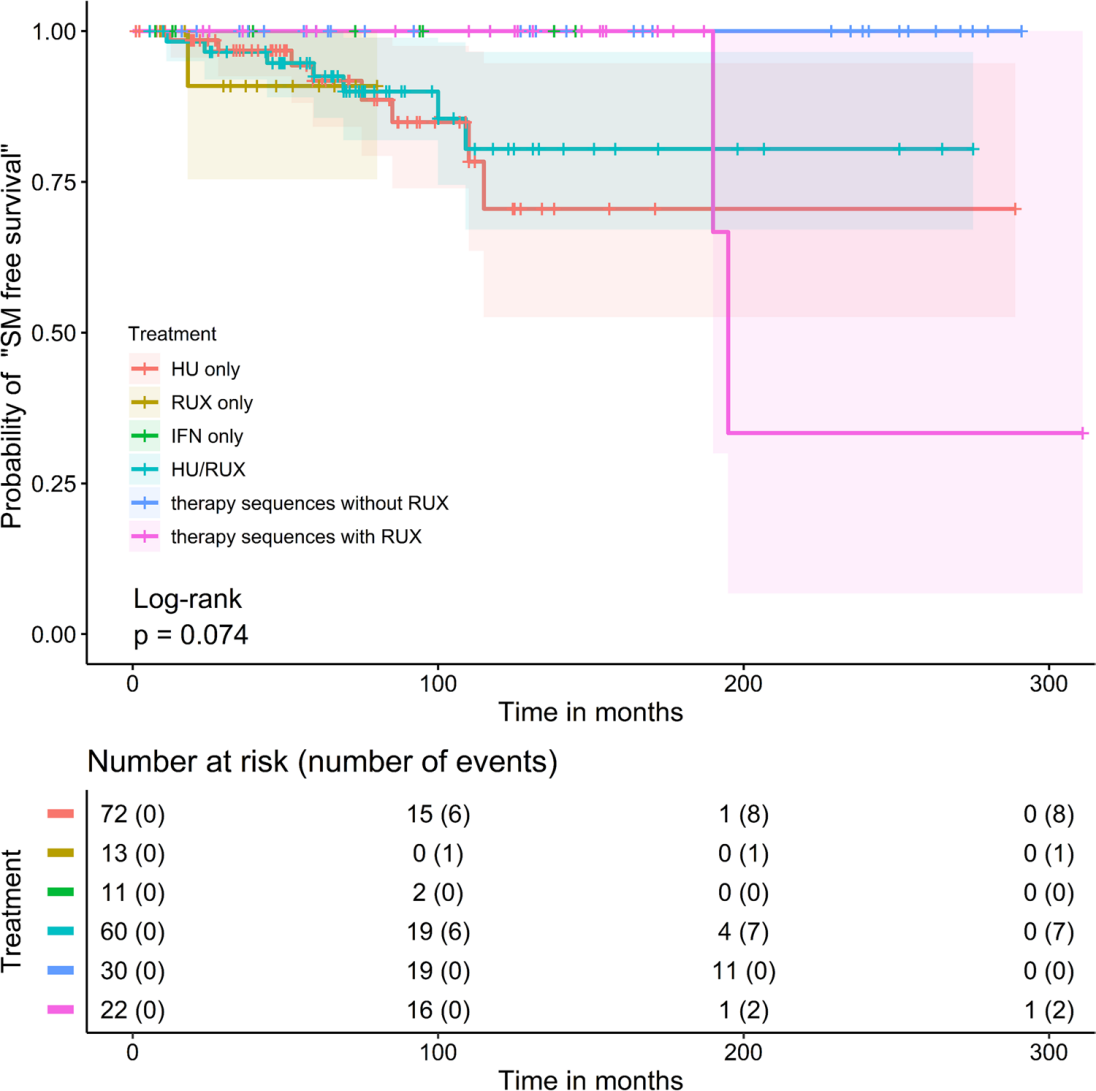
Probability of “secondary malignancy (SM) free survival” in six subgroups with different therapy sequences in 208 PV patients with cytoreductive therapy. Six subgroups with different therapy sequences were formed. Overall, no statistically significant difference was found among the six subgroups in the incidence of secondary malignancies (*p* = 0.074)

In the log-rank test, the probability of “SM free survival” was not statistically different between the six subgroups with different treatment sequences (Fig. 3, *p* = 0.074).

## Discussion

For many years, there was no standard therapy for high-risk PV patients (defined by age older than 60 years and history of thrombosis [17]) who developed intolerance or resistance to hydroxyurea (HU) [19, 20]. Since the pivotal “RESPONSE 1” [9] and “RESPONSE 2” [10] trials, ruxolitinib (RUX) has been the only approved drug for this setting, associated with an increased rate of hematological response, greater reduction in spleen volume, and improvement in PV-associated symptoms compared with best available therapy (BAT). Recently, however, concerns have arisen regarding an increased risk of SM, particularly lymphoma, associated with RUX treatment in MPN patients, although these data are predominantly from studies of primary or secondary MF patients [11–14].

Two retrospective studies [21, 22] with large MPN cohorts including PV patients showed no association between the development of aggressive lymphoma and JAK inhibitor therapy. However, different JAK inhibitors were administered and the occurrence of SM other than lymphoma was not investigated in these studies. In 2019, Barbui et al. [15] showed an increased risk of NMSC in RUX-treated MPN patients in their retrospective nested case–control study, but only 17 PV patients with RUX treatment (= 0.9% of all study patients) were included. In the 5-year follow-up data of the “RESPONSE 1” study [24], an increased risk of SM was observed in the group of patients initially treated with RUX compared with the BAT and the crossover population (7 cases versus 4.1 and 4.5 per 100 patient-years, respectively). The number of NMSCs was also higher in the RUX group (5.1 cases per 100 patient years) than in the BAT (2.7 cases per 100 patient-years) and crossover populations (2.7 cases per 100 patient-years). Similarly, preliminary 5-year follow-up data from the “RESPONSE 2” study [25] also showed an increased rate of NMSC in the RUX-treated and crossover populations (exposure-adjusted rates 2.7 and 2.9, respectively) compared with BAT (exposure-adjusted rate 1.9). However, a statistical comparison regarding the occurrence of SM or NMSC between the study arms with or without RUX treatment was not performed for either study.

To investigate the risk of secondary malignancies (SM) during long-term treatment with RUX in high-risk PV patients (> 85% had HU intolerance or resistance), we performed this retrospective “real-world” study. Only 5.9% (17/289) of patients were also enrolled in the two “RESPONSE” trials. The median RUX treatment duration of 48.0 months was comparable to the 5-year follow up data from the “RESPONSE 1” [24] and “RESPONSE 2” [25] trials. An increased risk of secondary malignancies was not observed in RUX-treated patients both in the analysis of all 289 patients or in the 208 patients who received cytoreductive therapy. In addition, multivariate analysis revealed no evidence of increased SM risk with RUX administration but only for patients with older age at PV diagnosis. No patient died due to RUX-associated SM.

However, the increased NMSC rate was striking and comparable to the prospective data from the “RESPONSE” trials and the retrospective study by Barbui et al. [15]. This is also consistent with the recently published data of Stegelmann et al. [23], who observed an increased risk of NMSC in HU-treated MPN patients of all subtypes in a prospective non-interventional study. Furthermore, in a long-term follow-up analysis of the “COMFORT 2” trial [13], an increased incidence of NMSC was observed in MF patients treated with RUX, and an association with HU pretreatment was discussed by the authors. Of note, almost two-thirds (63.6%) of NMSC in our study occurred in patients receiving RUX after HU pretreatment. Therefore, it is most likely that there is a correlation between the development of NMSC and HU pretreatment.

In summary, our data do not show an increased risk of secondary malignancies, particularly lymphoma, in PV patients treated with ruxolitinib. Therefore, in high-risk PV patients with HU intolerance or resistance, concern about developing SM does not appear to be a major factor preventing ruxolitinib therapy. However, our data indicate an increased risk of NMSC with RUX therapy, especially in the case of HU pretreatment. Therefore, in the future, the evolution of such malignant skin lesions in PV patients treated with RUX should be identified at an early stage by regular dermatological monitoring.

## Data Availability

The data that support the findings of this study are available from the corresponding author upon reasonable request.
